# Analysis of the visual evoked potential in anesthesia with sevoflurane and chloral hydrate (Variability of amplitudes, latencies and morphology of VEP with the depth of anesthesia)

**Published:** 2013-06-25

**Authors:** AM Ghita, D Parvu, R Sava, L Georgescu, L Zagrean

**Affiliations:** * University Emergency Hospital Bucharest, Romania; ** "Carol Davila" University of Medicine and Pharmacy, Bucharest, Romania

**Keywords:** visual evoked potentials, sevoflurane, chloral hydrate

## Abstract

The visually evoked potential (VEP) is an electrical signal generated by the occipital cortex in response to light stimulation of the retina. The clinical importance of the VEP consists in the diagnosis of optic nerve diseases and others ocular diseases. For experimental studies of VEP in experimental animals anesthesia is frequently required. Our study sought VEP changes depending on the type and depth of anesthesia.

Methods: this study evaluated VEPs in 20 Wistar rats under two anesthetics. Ten rats were anesthetized with sevoflurane and ten rats with chloral hydrate.

Results: The amplitudes, latencies and morphology of the VEP varied with the depth of anesthesia. The latency of VEP increases with the depth of anesthesia and the amplitude of the waves becomes more positive once the anesthesia decreases under sevoflurane and more negative under chloral hydrate. The variability of VEP was different under the two anesthetics with greater peak latencies under sevoflurane than under chloral hydrate at the same depth of anesthesia.

In conclusion: it is important to know the influence of the anesthetic and the depth of anesthesia over VEPS, because they may constitute a confounding factor in studying VEP in different diseases of optic nerve or eyeball.

## Introduction

The visual evoked potentials (VEP) are potential candidates for the monitoring of anesthesia because they reflect real-time changes in anesthetic depth, as well as the structural integrity of the eye [**1-3**]. In our study, we have comparatively analyzed VEP obtained at the induction of anesthesia with sevoflurane, respectively anesthesia with chloral hydrate, the purpose being that of identifying the changes that appeared according to the depth. The changes in latency and amplitude of the main peaks of the visual evoked potentials under sevoflurane (an inhaled anesthetic used in the general anesthesia of the children and adults) and the relationship between these changes and anesthetic depth measured through MAC (maximum alveolar concentration), as well as through the spectral analysis of the electroencephalographic recording are less known [**4,5**]. We have chosen the comparison between the anesthesia with sevoflurane and chloral hydrate because the literature regarding VEPs in anesthesia with chloral hydrate is rich and offers a good reference for the comparison of newer anesthetics [**6**]. 

 Surgeries for treatment of the tumors or vascular lesions localized along the visual pathways have a high risk of causing a visual dysfunction. Parasellar tumors or aneurisms, tumors in the temporal or occipital lobe, intraorbital tumors are all in close proximity to the visual pathway and resection surgeries endanger the pathways surrounding them [**7**]. 

 An accurate method for the real-time monitoring of the visual function would aid in taking intraoperative decisions regarding the radicalism of the excisions and the maneuvers done near the visual apparatus. Moreover, the VEPs could also aid in interpreting the changes that appear at the level of the primary and secondary visual fields in response to visual stimulation. Through the spectral analysis of the EEG signal, singular parameters such as the spectral edge frequency 90% (SEF 90) and the median frequency (MF) can be defined. The spectral edge frequency and the median frequency is the frequency under which 90% and respectively 50% of the EEG power spectrum is localized. Moreover, it has been suggested that SEF can be used to control the administration of the anesthetics through direct feedback and also to maintain a constant level of depth [**8**]. Thus, the SEF and MF can be used as quantifiers of anesthetic depth, both in chloral hydrate and in sevoflurane anesthesia. In interpreting the visually evoked potentials, we must take into account a series of elements that can lead to VEP modifications: ischemia, hypoperfusion, hypotension, hypothermia, increased intracranial pressure, important electrolytic modifications. All these can lead to the alteration of VEP and to a false interpretation of the results [**9-11**]. 

 In cases when VEP intraoperative monitoring is required, the most frequently used anesthetics are sedative-hypnotic drugs (benzodiazepines, barbiturates) and intravenous anesthetics (opioids, ketamine, propofol), because they allow better VEP interpretation. VEPs associated with deep anesthesia with chloral hydrate have more attenuated or absent negative compounds and the positive components of waves and the latencies of all peaks are greater [**6**]. A better knowledge of the changes induced by inhalation anesthetics on the VEP and their correlation with other parameters of anesthetic depth may allow their use in optimum intraoperatory conditions.

## Materials and methods

We conducted the experiments on 20 male Wistar rats, aged 3 months and weighing around 300 g, distributed randomly in the two study groups. The first group was assigned to choral hydrate anesthesia, while the second group was assigned to sevoflurane anesthesia. The animals were kept in the biobase of “Carol Davila" University of Medicine and Pharmacy, in transparent plastic boxes, with free access to water and food, the habitat temperature being of 23 degrees Celsius. The visually evoked potentials were recorded through wire implants. Chronic implantation was done under general anesthesia with chloral hydrate injected intraperitoneally (0.4g/kgc), after efficient anesthetic levels had been reached. The rat skull was fixed by a sterotaxic immobilization device. The electrodes were fixed directly to the skull without using adjacent substances. The material used to make the electrodes was the nichrome wire (ni80cr20, diameter of 0.15 mm). 3 electrodes were used; 2 were active electrodes and positioned 6 mm posterior and 4 mm laterally (left and right) from the bregma (corresponding to the visual area or area 17) and one reference electrode was placed 7 mm anterior to the bregma (corresponding to the olfactory bulb). 

 In the first stage of implantation, we performed a median incision across the skull and cleaned the cranial area of adjacent tissues. Afterwards, we burred 2 trephinations for each electrode, leaving a bony bridge of 1 mm between them, to which the electrode will be anchored. 

**Fig. 1:**
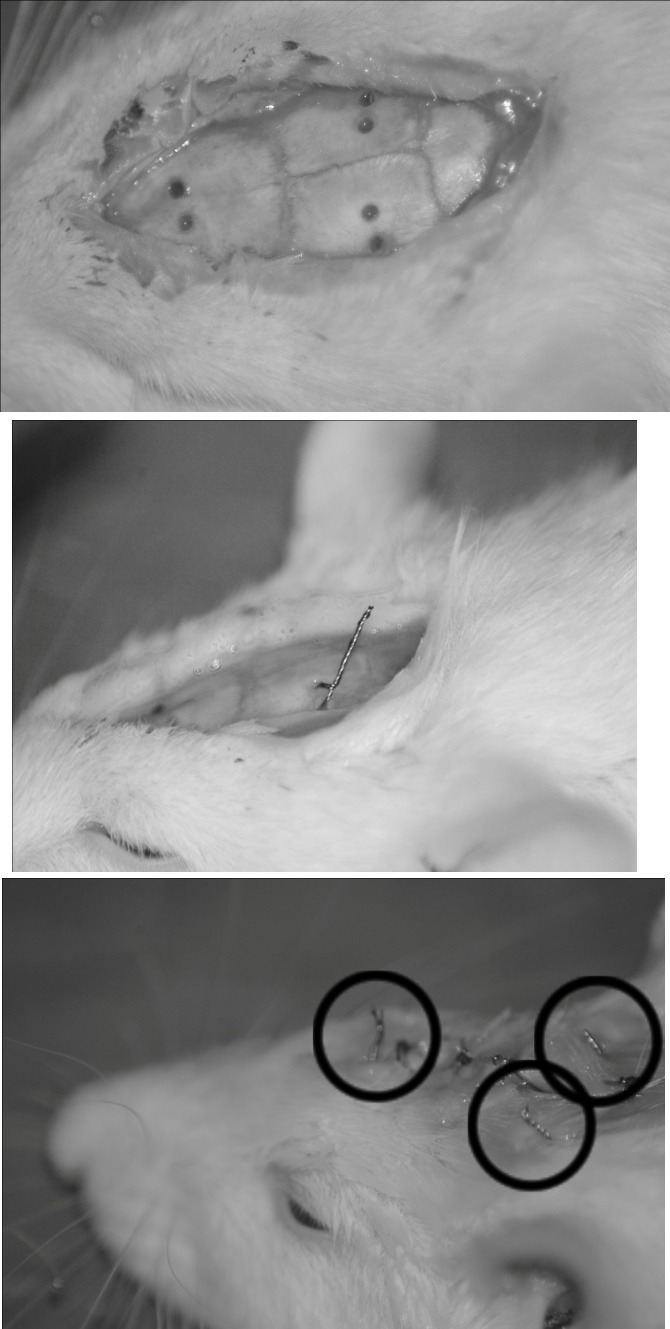
The stages of electrodes implantation in a Wistar rat

The animals were allowed to recover a week after implantation, after which we began recording VEPs under chloral hydrate and sevoflurane anesthesia. In the chloral hydrate group, anesthesia was induced by intraperitoneal injection (0.4g/kgc) and we recorded VEPs successively from the onset of anesthesia, to greater anesthetic depth and to awakening. In the sevoflurane group, induction of anesthesia was done at a sevoflurane concentration of 4 minutes using an oxygen vaporizer was used. VEP recording was done after an accommodation period of 15 minutes, at the following minimum alveolar concentrations (MAC): 3%, 2.5%, 2.2%, 2%, 1.8%, 1.6%, 1.4%, 1.2%, 1%, 0.8%, 0,6%. All the recordings were made in a soundproof darkroom. VEPs were evoked by a flash light stimulus that was placed at 1 cm below the eyes, in a 45 degrees angle, superior to the horizontal plane of the eye. The contralateral eye was covered with an opaque fabric. The flash generated light at a frequency of 30 stim/minute, with a length of the stimulus of 0.015s. The duration of the VEP recordings was of 5 minutes and the VEP was obtained through the mediation of 300 signals. 

### Obtaining and analyzing the data

The electrodes were connected to the BIOPAC MP150 system and the data acquired with the Acqknowledge 4.2. program. The measured impedance of the electrodes was below 100Mω. The signal was amplified 5000 times and the artifacts and the interferences have been eliminated with a 1-100Hz crossband filter. Data was recorded at 1000 frames per second. The VEP waveform has been reported to have many peaks, but we only analyzed 4 peaks that appeared most constantly in our study. The positive peaks are noted P and the negative peaks are noted with N. The amplitudes and latencies of the 4 VEP peaks were calculated. The spectral analysis of the EEG recording was done with Acqknowledge 4.2. program. We obtained the MF and the SEF for a 5 minutes’ recording by the mediation of the values calculated in 4 seconds periods. MF and SEF was calculated within both the chloral hydrate and sevoflurane group as a measure of anesthetic depth.

 For the sevoflurane anesthesia, we have applied the linear regression in order to research the existence of a linear correlation between MAC and MF or SEF, the latency of the visually evoked potentials and MAC, the period between the latency of the N1-P1, N2-P1 peaks and MAC. Moreover, we have calculated the amplitude of N1-P1 and N2-P1 periods at different MACs. We have also used the linear regression in the chloral hydrate anesthesia, in order to highlight the correlations between the latency of N1, P1 and N2 peaks, the difference between the latencies of N1-P1, N2-P1 peaks, and the difference between the latencies of the peaks, the amplitude of these periods and MF or SEF. 

## Results

We recorded visual evoked potentials at various anesthetic depths. The depth of the anesthesia has been quantified by MF and SEF. We sought to establish a correlation between the depth of the anesthesia and the median frequency, respectively the spectral edge frequency has been done in the case of light and moderate anesthesia. 

 Under sevoflurane anesthesia, we found a high degree of correlation between the MAC and the MF and SEF, when the MAC value is below 2%. In deeper sevoflurane anesthesia (MAC 2-3%) and in chloral hydrate anesthesia, the appearance of the “burst-suppression" phenomena [**6,12-14**] randomly modifies the median frequency. Thus, in deep anesthesia, the MF and SEF are not reliable indicators of anesthetic depth and the measurement off the burst-suppression ratio is necessary to correctly evaluate anesthetic depth. Our results are presented for all the degrees of depth, using MAC as a quantifier for anesthetic depth in sevoflurane anesthesia. 

**Fig. 2:**
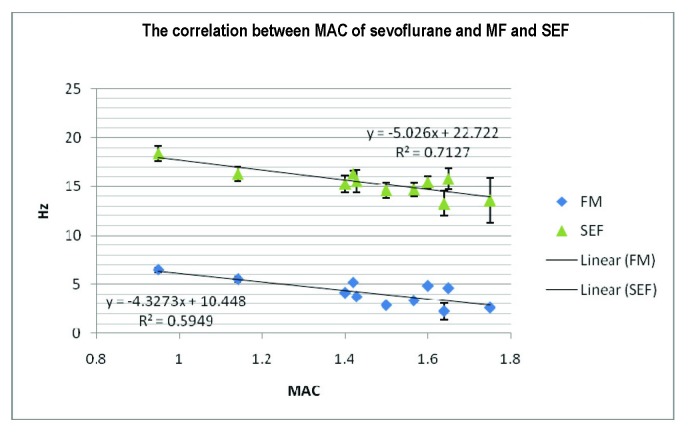
The correlation between the minimum alveolar concentration of sevoflurane and the median frequencies and the spectral edge frequencies over 10 recordings. The correlation between MAC and SEF is strong, with a Pearson’s coefficient of r = 0,843 at MAC values between 0.8-1.8%. The correlation between median frequency and MAC is lower (r = 0,77).

 In the range of MAC values between 0,8% and 1,8%, there is a strong correlation between the anesthetic depth as measured by MAC and SEF. The level of correlation of MF with MAC is lower than that of SEF, thus SEF proves to be a more reliable indicator of anesthetic depth than MF (**Fig. 2**).

 The visually evoked potentials in sevoflurane anesthesia 

 Changes in morphology of the visual evoked potential 

**Fig. 3:**
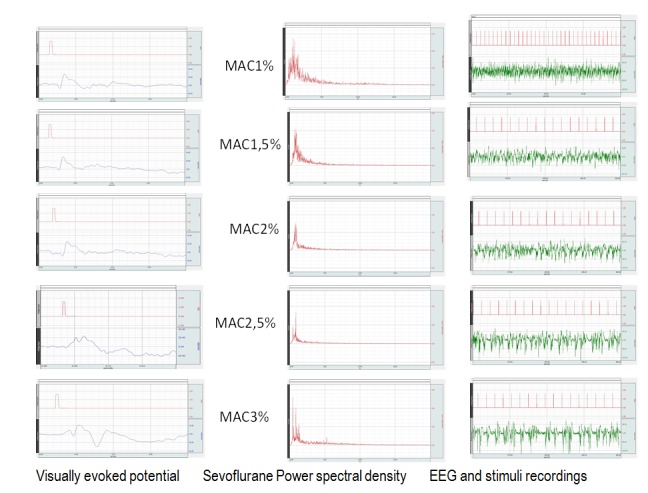
Changes in morphology of the VEP, the EEG power spectrum and the EEG recording under flash stimulation. Note the classic aspect of the VEP wave in superficial anesthesia (first recording, MAC 1%). An increase of the spectral power along with the waning of anesthesia is observed in the middle column. A decrease of the suppression periods to the point of their disappearance is observed on the EEG recording along with the waning of the anesthesia.

 The changes in morphology of the VEP can be noticed in Fig. 3. Accordingly, in deep anesthesia, N2 wave is better expressed in comparison to wave P1. With lowering anesthetic levels, an increase in the amplitude difference between N1 and P1, respectively N2 and P1 takes places, together with a decrease of MAC and the increase in the median frequency and the spectral edge frequency. With a better expression of wave N1 and an increase in the amplitude of wave N2, the morphology of the VEP changes so that in superficial anesthesia the “classic" aspect of the VEP is highlighted, with well-defined N1 and P1 waves, followed by an ample N2 wave. 

 Changes in latency of the visual evoked potential under sevoflurane 

 The latencies of the peaks of the VEP vary with anesthetic depth. We calculated the averages for the latencies of peaks N1, P1 and N2 and observed that, for increasing MAC values, there is an increase in the latency of peaks N1 and P1 and a decrease in latency of peak N2 (**Table 1**). 

**Table 1.**
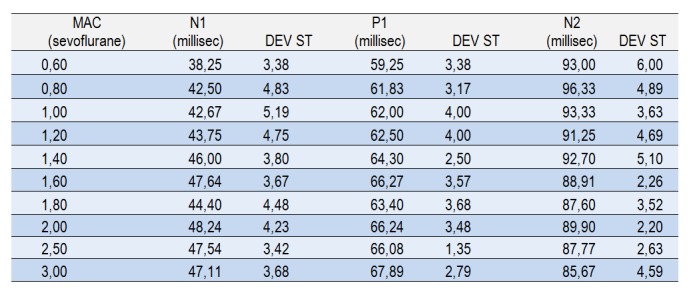
The average of the latencies and the standard deviation (DEV ST) corresponding to peaks N1, P1 and N2 at different degrees of anesthetic depth quantified through the MAC. Together with the depth of the anesthesia the latency of N1 and P1 waves increases, a good index of statistical correlation (R = 0,9353 and R = 0,9126 respectively) is noticed.

**Fig. 4:**
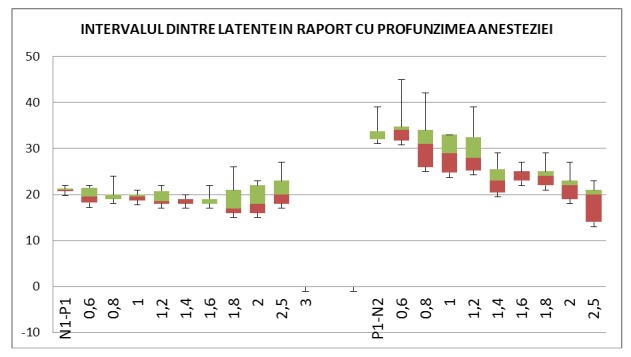
The variation of latencies expressed as N1-P1 and N2-P1 with depth of anesthesia quantified through the MAC. The variation of latencies is calculated by subtracting the previous latency out of the following latency. The data marked by squares shows the variation of N1-P1 at different degrees of and a good correlation score (R= 0,8096). The data marked by diamonds shows the variation of N2 and P1 latencies at different degrees of anesthetic depth and a good correlation score (R = 0,8383).

 Amplitude changes of the visual evoked potential in sevoflurane anesthesia 

 The amplitude of the VEP waves modifies during the anesthesia. The amplitude of the wave in absolute value cannot be directly quantified because the recording system sets the value “0" by summing up the currents from the starting moment of the recording. Thus, the amplitude of the waves will be expressed as “peak-to-peak".

**Table 2.**
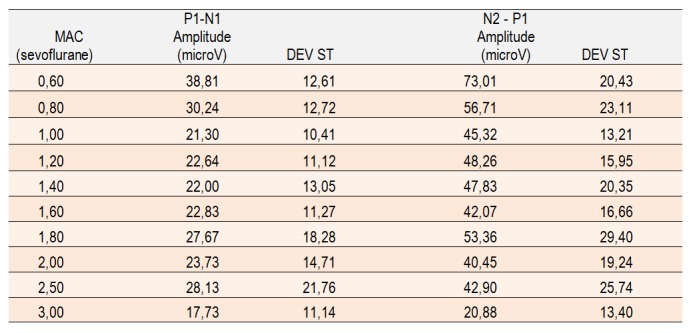
The amplitudes of P1-N1 and N2-P1 in different degrees of anesthetic depth (MAC).

 During the waning of anesthesia, the amplitude of wave P1 compared to N1 wave increases (**Table 2**), as does the amplitude of wave N2 compared to wave P1. The changes in amplitude of N1-P1 do not have a powerful statistical correlation but they can be observed in all of the recordings, however, the N2-P1 amplitude change is correlated with MAC (r=0,892). In light anesthesia, we noticed an increase in the positive components of the VEP through the increase of P1-N1 and N2-P1 amplitudes.

 The visual evoked potentials under chloral hydrate anesthesia 

 Morphology changes of the visual evoked potential under chloral hydrate 

**Fig. 5:**
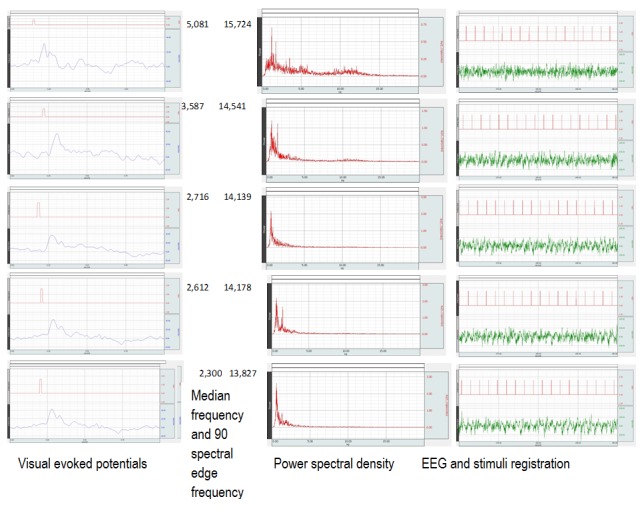
Changes in VEP morphology, the EEG power spectrum and the EEG recording while applying light stimuli. Some examples of visual evoked potentials at different MF and SEF are presented.

 The morphology of the VEP changes in chloral hydrate anesthesia, similarly to the descriptions available in literature. In deep anesthesia, wave N2 is well expressed, while during the waning of anesthesia, the morphology gradually changes, with a better expression of wave N1 compared to wave P1 and a decrease in amplitude of wave N2 wave compared to wave P1. The rise of MF and SEF (lower anesthetic levels) highlights an increase in the amplitude of P1 wave compared to N1 wave, and a decrease compared to wave N2. Thus it appears that in light anesthesia, the visual evoked potential is better represented (**Fig. 5**). 

 A change in the morphology of the visual evoked potential with a better evidencing of N1 and P1 waves in deep anesthesia and of N2 wave in superficial anesthesia can be observed in Fig. 5. Moreover, an increase in the amplitude of P1 wave and a decrease in the amplitude of N2 wave can be observed while the anesthesia wears off. There is a correlation between the morphology of the wave and the depth of the anesthesia. An increase in spectral power can be observed in the middle column, while the anesthesia wears off. Likewise, a decrease of the suppression periods can be observed on the EEG recording, and their disappearance can be noticed while the anesthesia wears off. 

 Changes in latency of the visual evoked potentials under chloral hydrate anesthesia 

 The latency of the waves changes with anesthetic depth. We evaluated the relationship between anesthetic depth, expressed through MF and SEF, and the latency of N1, P1 and N2 waves (**Table 3**). In our study, the rise of the MF and the SEF is accompanied by the decrease in the latency of all VEP peaks. By applying linear regression techniques, we observed a strong correlation between the MF and latencies of peaks N1 (r=0,854), P1 (r=0,676) and N2 (r=0,781) and a strong correlation between SEF and peaks N1 (r=0,870), P1 (r=0,675) and N2 (r=0,836). 

**Table 3.**
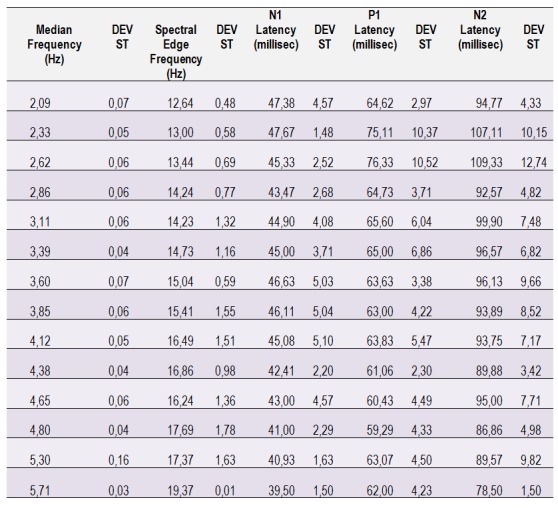
The latency of peaks N1, P1 and N2 of the VEP at different depths of anesthesia. The average of the median frequencies, the spectral edge frequencies and the latencies for each 0,5 Hz period of the median frequency between 2 and 6 Hz, has been calculated.

 The difference between the periods of the latencies of two consecutive waves is equivalent to the interval between the latencies of these waves and is a good descriptor of latency variation at different anesthetic depths. With greater depth of the anesthesia, there is a slight increase in the period between P1 and N1 waves. In addition, the difference between N1 and P1 latencies negatively correlate with the MF and the SEF; the difference between the latency of N2 and P1 waves correlate weakly while the anesthesia wears off. 

**Fig. 6:**
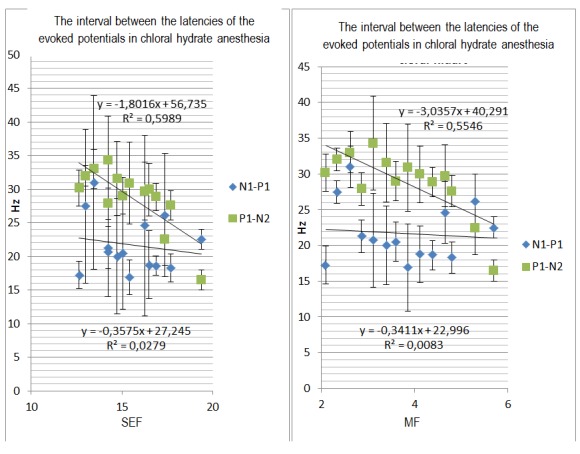
a) Correlation between the variation of the N1-P1 and N2-P1 intervals and the spectral edge frequency r=0,1643; r=0,7738 respectively; b) Variation of the N1-P1 and N2-P1 intervals with the spectral edge frequency; r=0,722; r=0,0912 respectively.

 Changes in amplitude of the visual evoked potential under chloral hydrate 

 The amplitude of the waves’ changes in relation to the depth of the anesthesia (**Table 4**), our data being similar to the data in the specialty literature. A slight modification in the value of the amplitude of N1-P1 at lower levels of anesthesia can be noticed, a variability without a statistical significance. Instead, the P1-N2 change in amplitude has a higher variability with depth of anesthesia, negatively correlated with the MF (R=0,7459) and the SEF (R=0,7412). In addition, it can be stated that at lower anesthetic levels there is a significant increase in the amplitude of N1-P1 and an important decrease in the amplitude of P1-N2.

**Table 4.**
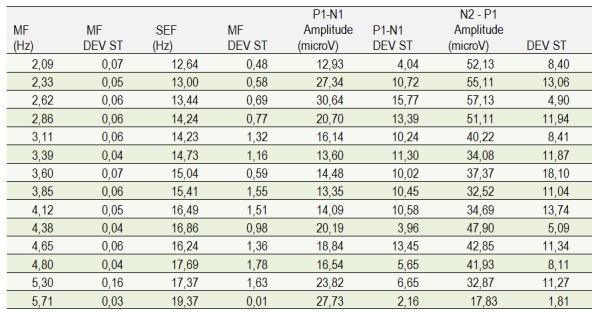
The value of the amplitude peak-to-peak for P1-N1 and N2-P1 in different degrees of depth of anesthesia, quantified by MF and SEF

 Comparison between VEP under sevoflurane and chloral hydrate anesthesia 

 According to the data presented, we identified multiple similarities and differences between the VEP polymorphism in sevoflurane and chloral hydrate anesthesia. 

**Fig. 7:**
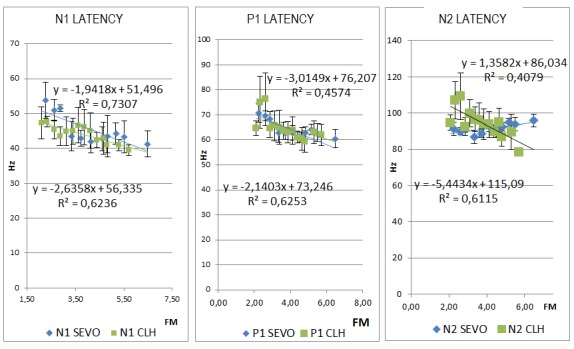
Comparison between the latency of peaks N1, P1 and N2 at different degrees of anesthetic depth under sevoflurane (SEVO) anesthesia. a) A similar change to that of the latency of peak N1 is observed under both types of anesthesia. b) The slope of P1 latency variability is highlighted under chloral hydrate and it is attenuated under sevoflurane (p>0,01). c) The tendency of variation of the peak N2 latency is opposed in sevoflurane in comparison with chloral hydrate anesthesia

**Fig. 8:**
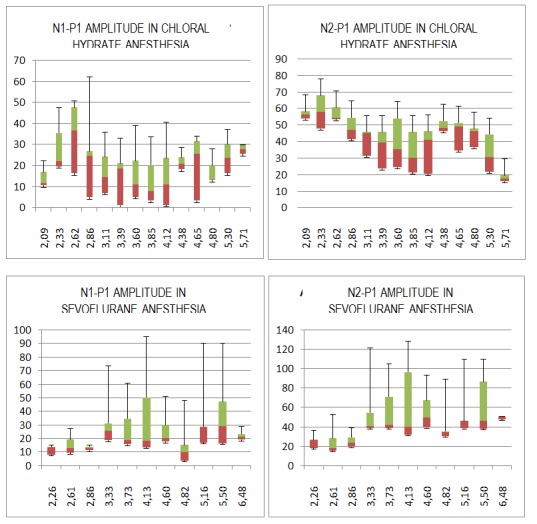
Box and plots for the amplitude of VEP N1-P1 and N2-P1 waves in chloral hydrate and sevoflurane anesthesia according to the EEG median frequency. For the chloral hydrate anesthesia, N1-P1 and N2-P1 median amplitude were measured for each frequency interval of 0,25Hz, between 2-5 Hz and for each interval of 0,5 Hz, between 5-6 Hz. For the sevoflurane anesthesia, the frequency interval was of 0,5 Hz between 2,5 - 3 Hz and of 0,25 Hz between 4.5 - 5 Hz.

 Focusing on peak N1 (**Fig. 7**), its latency increases and amplitude (N1-P1 “peak-to-peak" amplitude) (**Fig. 8**) decreases together with the depth of anesthesia, both under chloral hydrate and under sevoflurane. This aspect is given by the latency in the presence of the nervous stimulus on the optical paths together with the suppression induced by the anesthesia. This tendency can be explained through the slowing of neuronal conduction under anesthesia. 

 A second tendency of variability of the VEP refers to the latency of P1 wave. P1 latency rises together with the depth of the anesthesia, the variability of the latencies correlating with the depth of the anesthesia measured by the MF or the SEF under both types of anesthesia (**Fig. 7**). However, differences are observed as far as the statistical correlation slope line is concerned, thus the latency slope of P1 wave varies more amply in chloral hydrate anesthesia than in sevoflurane anesthesia. 

 A major difference between chloral hydrate anesthesia and the sevoflurane anesthesia is observed in the latency of N2 wave (**Fig. 7**). Under chloral hydrate, the tendency of the latency is to increase with depth of the anesthesia, a change that is statistically correlated both for the MF (r=0,7819) and for the SEF (r=0,6386). Moreover, under sevoflurane anesthesia there is a decrease of the latency with an increase in the depth of the anesthesia (MF, SEF, MAC) observed in all the recordings (r=0,638 for MF).

 Another important point is that the amplitude of wave N2 increases with respect to wave P1 (“peak-to-peak" amplitude) (**Fig. 8**), which modifies according to the depth of the anesthesia. There is an increase in the N2-P1 amplitude while the sevoflurane anesthesia wears off, statistically correlated with the MF (r=0,564). However, the N2-P1 amplitude under chloral hydrate anesthesia negatively correlates with the depth of the anesthesia, r=0,7459 for the MF and r=0,7412 for the SEF. 

## Conclusions

In our study, the visual evoked potential varies under both types of anesthesia. The results under chloral hydrate anesthesia are similar to data in the literature. The anesthetic depth under chloral hydrate anesthesia has been evaluated through MF and SEF. According to previous data in the literature, both parameters correlate with anesthetic depth. Our study could not monitor the serum levels of chloral hydrate during anesthesia. The data obtained showed that, during anesthesia, the morphology of the waves changed, in the sense that the amplitude of the peak-peak waves was lower in deep anesthesia, so that the whole set of waves of the visually evoked potentials was negative. At lower anesthetic levels (median frequency 3-5 Hz and spectral edge frequency 15-17 Hz), there is a light increase in the amplitude of wave N1 with respect to wave P1, and a significant decrease of the N2 amplitude with respect to P1 wave, statistically correlated with MF and SEF. In addition, in lighter anesthesia under chloral hydrate the P1 wave is better evidenced with respect to N1, but with a reduction with respect to the amplitude of wave N2. 

 The sevoflurane anesthesia produces changes contrary to chloral hydrate anesthesia. If the amplitude of N1-P1 wave changes slightly with a small decrease but uncorrelated statistically with the MF and the SEF, the change of N2-P1 wave, measured as peak-to-peak shows a rise at lighter anesthetic levels, being positively correlated with the MF. This change of the N2-P1 amplitude in the sevoflurane anesthesia is different from chloral hydrate anesthesia, in which a reduction takes place together with the rise in MF. 

The most important change regarding chloral hydrate anesthesia is the increase in the latency but also of the period between the latencies along with the increase in the depth of the anesthesia. In addition, in low depth anesthesia (median frequency of 5,29) the latency of peak N1 is of 39,6ms and, in the deep anesthesia (median frequency of 2,18) the latency of peak N1 is of 45,61ms. This increase in latencies is present for both P1 and N2 in the chloral hydrate anesthesia and it is statistically correlated with the MF and SEF. 

 The sevoflurane anesthesia was evaluated by two major parameters: the MAC and the MF and SEF according to periods of 4 sec. The depth of the anesthesia correlates with the MF until it reaches the value of 2% MAC. There is a statistical linear correlation between the sevoflurane concentration (0,8 and 2% MAC) and the MF and the SEF. Over this value, the increase in sevoflurane concentration leads to the appearance of “burst-suppression" episodes with progressively high periods of suppression. The median frequency and the spectral edge frequency for MAC, which are higher than 2%, do not have a statistical correlation. The analysis of the potential was done according to the sevoflurane concentration (0,6-3%) as well as to the median frequency and the spectral edge frequency, to MAC concentrations of 2%. It can be noticed that, through both parameters, once the depth of the anesthesia rises, the morphology of the waves of the visually evoked potential and the amplitude of the visually evoked potentials change differently from the chloral hydrate anesthesia. Tracking the latency of the waves, an increase in the latency of N1 and P1 waves is noticed together with the increase in the depth, monitored by MAC but also by the median frequency and the spectral edge frequency. The variability of the latency of N2 wave, which decreases according to the depth of the anesthesia, has a behavior that is different from that under chloral hydrate anesthesia. An additional observation is also the diminution of the period of time between the latencies, respectively N1-P1 and N2-P2, together with the increase in the depth of the anesthesia.

## Discussions

The change of the VEP under sevoflurane shows an increase in the latency of wave N1 and a slight increase of latency P1 with the increase in the depth of the anesthesia. These changes are in accordance to those observed under other anesthetics. In addition, we can state the fact that the anesthesia suppresses nervous conduction through the optical paths, determining a late cortical evoked response to the visual stimuli (from 38,25ms to a MAC of 0,6% to 47,11ms to a MAC of 3%). It seems that N1 and P1 waves are generated at the level of the main cortical visual areas and N2 and P2 waves are generated at the level of the secondary cortical areas [15-16]. The duration between the appearance of N1 wave and that of P1 wave slightly decrease with the depth of the anesthesia; the decrease being more important for N2-P1 interval (33,75ms for MAC 0,6% and 17,77ms for MAC 3%). This change in the duration of N1-P1 and P1-N2 intervals shows an increase in the intracortical communication under sevoflurane anesthesia. In comparison to chloral hydrate anesthesia, together with the nerve conduction depression, it produces a decrease in the intracortical communication, with an increase in the latencies of all the waves of the visually evoked potential, as well as in the duration of N1-P1 and N2-P1 intervals. The change of the different cortical answer in the visual stimulation in sevoflurane anesthesia is still under investigation, in order to better understand the way in which sevoflurane induces the anesthesia. 

 While tracking the peak-to-peak amplitude for N2-P1, it can be observed that together with the increase in the latency of N2 wave, a diminution of it takes place both in the sevoflurane anesthesia and in the chloral hydrate anesthesia. The major difference is that in the case of chloral hydrate anesthesia, the amplitude of N2-P1 wave and that of the latency is maximum in deep anesthesia and, in the case of sevoflurane anesthesia, it is maximum in superficial anesthesia. 

 To conclude, chloral hydrate anesthesia and the sevoflurane anesthesia induce similar changes in the case of N1 and P1 peak latencies and different changes of wave N2. This phenomenon shows that both anesthetics produce a delay of the visually evoked signal, but at the cortical level, the processing of the visual signal is different in the chloral hydrate anesthesia compared to the sevoflurane anesthesia. In addition, it can be said that the chloral hydrate anesthesia determines a delay in the conduction of the visual stimulus in all the structures involved, in generating the visually evoked potential: visual paths, primary and secondary areas. Sevoflurane determines a delay in the conduction of the visual stimulus to the cortex, but at the cortical level, it increases the transmission between the different areas that evoke the light stimulus. 
